# Genetic and Clinical Progression of MYSM1 Related Bone Marrow Failure into Myeloid Malignancies: Case Series and Review of Literature

**DOI:** 10.46989/001c.138314

**Published:** 2025-06-16

**Authors:** Alfadil Haroon, Syed Osman Ahmed, Chokri Ben Lamine, Mahmoud Aljurf, Hazzaa Alzahrani

**Affiliations:** 1 Adult Hematology, stem cell transplant and cellular therapy section, Cancer Center of Excellence, King Faisal Specialist Hospital and Research Center, Riyadh, Saudi Arabia

**Keywords:** MYSM1 mutation, bone marrow failure, Hematopoietic stem cell transplantation, Acute myeloid leukemia, Myelodysplastic syndrome

## Abstract

MYSM1, located on chromosome 1p32.1, encodes histone H2A deubiquitinase, a transcription regulator involved in DNA damage response. Biallelic MYSM1 variants are linked to rare bone marrow failure syndromes, presenting with cytopenia, B-cell deficiency, hypogammaglobulinemia, and developmental abnormalities. We report four cases of MYSM1 mutations progressing from marrow failure to MDS or AML within 9–10 years. Genetic abnormalities, including TP53 mutation and chromosomal anomalies, suggest clonal evolution. Hematopoietic stem cell transplantation achieved remission in two patients with adverse cytogenetics. Further research is needed to refine management strategies and assess long-term outcomes in MYSM1-associated marrow failure and MDS.

## Introduction

Inherited bone marrow failure (IBMF) increasingly being recognized with the wider availability of next generation sequencing (NGS) platforms and greater awareness of these conditions. especially in the setting of consanguinity where the causal mutation usually resides within the easily tractable autozygome^1^

BMF secondary to mutations in Myb-like SWIRM and MPN domains (MYSM1) were first identified in two siblings who presented with anemia and facial dysmorphism,^2^ and have been more widely characterized by subsequent studies.

MYSM1is located on chromosome 1p32.1 and encodes histone H2A deubiquitinase, which is composed of 829 amino acids and functions as a chromatin-binding transcriptional regulator causing histone H2A (H2AK119ub) deubiquitination.^3^ additionally, it regulates termination of DNA damage responses^4^ Recently, biallelic variants in MYSM1 have been reported to lead to a rare BMF in human.^5,6^ affected patients showed hematological features of BMF such as leukopenia, granulocytopenia, thrombocytopenia, B-cell deficiency, severe anemia, and hypogammaglobulinemia. Non hematological abnormalities, such as developmental delay, short stature, facial dysmorphy, and microcephaly have been described in patients with MYSM1 mutation.^2,5–12^ Targeted deletion of murine MYSM1 results in severe hematopoietic defects associated with an early block of B-cell development; dysfunction of stem cell maintenance, self-renewal, and differentiation; and natural killer (NK) cell defects^13–15^ In total, 12 patients and 5 variants of MYSM1 mutation have been reported to date. We report four cases who progress to myeloid malignancy (MM), including the 10-years follow-up update for the first two patients who were diagnosed with BMFS.

**Table 1.** represent patients characteristics

	**Family 1Case 1**	**Family1 Case 2**	**Family 2 Case 3**	**Family 2 Case 4**
Age at diagnosis	4 months	15 months	Since birth	Since birth
Gender	female	male	male	male
Hematological presentations	Anemia	Anemia	Anemia	Anemia
Non-hematological features	Facial dysmorphism	Facial dysmorphism	non	non
Ethnicity	Arab	Arab	Arab	Arab
Initial diagnosis	BMFS	BMFS	DBA/BMFS	DBA/BMFS
Myeloid transformation	Hypoplastic MDS	Hypoplastic MDS	Hypoplastic MDS	MDS/AML
Age at MDS/AML Transformation	10 years	10 years	12 years	12 years
NGS/FISH	5q deletion CALR	TP53 mutation	Monosomy 7 trisomy 8	Monosomy 7
BM cellularity at initial diagnosis	10%	20%	5%	10%
BM cellularity at myeloid transformation	10%	30-40%	5%	100%
morphology at myeloid transformation	Megakaryocytic dysplasia	fibrosis erythroid dysplasia	fibrosis erythroid dysplasia	73% myeloblast
HSCT	Haploidentical	No-plan for Haploidentical	Haploidentical	no
Outcome	alive	alive	alive	Died

CALR: calreticulin, HSCT: hematopoietic stem cell transplant, DBA: Diamond Blackfan Anemia, NGS: next generation sequencing, FISH :Fluorescence In Situ Hybridization, BM:bone marrow

### Case 1

A 15-year-old female patient with BMFS caused by a mutation in MYSM1 presented initially at the age of 4 months with anemia. She remained transfusion-dependent till the age of 10 years, followed by a phase of spontaneous recovery. However, she again became transfusion-dependent with the onset of menarche. Bone marrow examination showed hypocellular marrow (10%) with features of MDS. NGS identified a CALR p.(p228s) mutation with a variant allele frequency (VAF) of 50%, and FISH showed mono-allelic loss of the EGR1 (5q31) gene.

She underwent haploidentical hematopoietic stem cell transplant (HSCT). The conditioning regimen consisted of fludarabine, cyclophosphamide, and total body irradiation-200 cGy and Anti-Thymocyte Globulin (Flu/Cy/TBI/ATG). Tacrolimus, mycophenolate mofetil and PTCy, were used for GvHD prophylaxis. Neutrophil engraftment was achieved by day +13 and platelet engraftment by day +20. Now 6 months post-HSCT, she remains fully chimeric. Notably, her pre-transplant distal renal tubular acidosis with severe hypokalemia resolved following HSCT.

### Case 2

A 14-year-old male was presented at 15 months of age with pallor with hemoglobin of 4.4 g/dL. Bone marrow examination showed marked erythropoiesis, decreased granulopoiesis and megakaryopoiesis, dysplastic erythroid precursors, and megakaryocytes with cellularity of 20%. He received monthly blood transfusions up to the age of 33 months and slowly became transfusion-independent thereafter. A recent bone marrow biopsy showed hypocellular marrow (~30-40%) with dyserythropoietic changes and a TP53 mutation. He is currently receiving PRBC transfusions every 2-3 weeks and has been started on eltrombopag. There is no MSD, and he is being considered for haploidentical HSCT.

### Case 3

A 19-year-old male initially presented with anemia at birth. He required PRBC transfusions in infancy and was stable until age 9 when his Hb declined, necessitating low-dose steroids. diagnosed with Diamond Blackfan Anemia (DBA). At 11, his hemoglobin dropped to 6.5 g/dL, requiring high-dose prednisone. At 12, he presented with pancytopenia and bone pain. Bone marrow biopsy revealed 5% cellularity. FISH showed the presence of 7q deletion and trisomy 8. In 3 months, he progressed to fibrotic hypoplastic MDS. Whole-exome sequencing confirmed a pathogenic MYSM1 mutation. DBA and other causes of BMFS were excluded. He received haploidentical HSCT at 12 years of age using Flu/Cy/TBI/busulfan as conditioning regimen and achieved full chimerism but developed chronic GVHD affecting the eyes and lungs. Family history revealed consanguinity, a sibling homozygous for MYSM1, and several relatives with severe anemia or neonatal death.

### Case 4

12 years same presentation as sibling Case 3, and diagnosed as DBA during infancy. Required PRBC transfusions and remained stable until the age of 9 years, when CBC revealed pancytopenia; bone marrow biopsy showed MDS transformation with monosomy 7. WES confirmed mutation in MYSM1. his clinical course complicated with recurrent infections including lung fungal infection, perianal abscess and necrotizing fasciitis. 3 months later he transformed to AML, BM biopsy was hypercellular marrow up to 100% with 73% myeloblast, received mini-flag chemotherapy. He passed away 3 months post induction chemotherapy due to septic shock

## Discussion

MYSM1 mutation variants are associated with BMFS and MDS, with phenotypic diversity observed across cases. To the best of our knowledge, 12 (including 2 of our patients) cases have been reported^2,5–12^ table (2). Most of the patients had presented with BMF characterized by anemia in 9 cases, bicytopenia in two-thrombocytopenia and leukopenia one pancytopenia accompanied by immune deficiencies such as B- and NK-cell abnormalities, low IgG and IgM levels. Non- hematological features include, developmental abnormalities like microcephaly, dysmorphic features, and neurodevelopmental delays. Infections included respiratory tract infections; 3 required HSCT for treatment. The results were variable; some patients survived after HSCT or spontaneous recovery. The findings underline the responsibility of MYSM1 in BMF syndromes, highlighting its phenotypic variability and how early genetic diagnosis may influence clinical decisions.

**Table 2.** summaries characteristics and outcomes of reported cases with MYSM1 mutation

**Authors**	**Case #**	**Gender**	**Consanguinity**	**Ethnicity**	**MYSM1 mutation**	**Hematological findings**	**Non-hematological findings**	**Immunological finding**	**Outcome**
Absultan et al, 2013	1	female	yes	Arab	c.1168G>T E390X	Anemia, BMF	Facial dysmorphism	Neutropenia, decreased B-,NK-cells, ,IgM level	Alive, transformed to MDS 6 months post haplo-HSCT
	2	male	yes	Arab	c.1168G>T E390X	Anemia, BMF	Facial dysmorphism	Neutropenia, decreased B-,NK-cells, ,IgM level	Alive , transformed to MDS Preparing for HSCT
LeGuen et al, 2015	3	male	yes	Turkish	c.1973A>G H658R	BMF (anemia, leucopenia)	Microcephaly, choanal atresia, bilateral deafness	Decreased B cells, T-cells ,CD4,	Alive, recovered a normal immunohematology phenotype
Bahrami et al, 2016	4	female	yes	Arab	с.1168G>T E390X	BMF (anemia, thrombocytopenia,lymphopenia, neutropenia)	Short stature and mild dysmorphic features cataracts, and neurodevelopmental delay	Decreased B-cells,CD21,CD4 ,IgM level	HSCT (42m) – alive
	5	male	yes	Arab	с.1168G>T E390X	BMF (anemia, mild thrombocytopenia, lymphopenia, moderate to severe neutropenia)	Short stature ,dysmorphic features cardiomyopathy ,neurodevelopmental delay	Decreased B-cells,CD21,CD4 IgM level	HSCT (23m) - alive
Ulirsch et al, 2019	6	Unknown	unknown	Unknown	c.1432C>T R478X	DBA (clinical data on individual patients are not provided)	not provided	not provided	not provided
Nanda et al, 2019	7	female	unknown	Kuwait	с.1168G>T E390X	BMF (anemia, leucopenia)	tricuspid regurgitation,developmental delay,intellectual disability	Neutrophil panniculitis,B-cell deficiency	Preparing for HSCT (5y at the moment of publication)
N. Li et al, 2020	8	female	no	China	c.1467C>A p.Y489X/c.399G>A(synonymous, splice defect - p.V108LfsX13)	BMF (anemia, leucopenia, thrombocytopenia)	No congenital abnormalities	reduced granulocyte,B,T,NK-cell and IgG	Preparing for HSCT (18m at the moment of publication)
Barhoom et al, 2021	9	female	yes	Iran	c.1965T>A Y655X	Anemia, thrombocytopenia, leucopenia	No congenital abnormalities	B-cell deficiency, low IgG	Alive 12y post somatic genetic rescue
Zhan et al, 2021	10	female	no	China	c.1432C>T R478X	Anemia, leucopenia	polydactylism with six fingers on the right hand, brain cyst	B and T-lymphopenia	not provided
Mantravadi et al, 2021	11	Unknown	unknown	USA (ethnicity unknown)	c.1843-1G>A	Pancytopenia	not provided	Was diagnosed with SCID	not provided
Sharapova et al, 2023	12	male	no	Belarus	c.412C>T R138X	Anemia, neutropenia, bone marrow failure	Growth retardation, chest deformity, cataract	Decreased B,NK-cells, IgG level	Alive
	13	male	yes	Arab	с.1168G>T E390X	Anemia -diagnosed as DBA	Non	No	Transformed to AML, died at age of 12y
	14	male	yes	Arab	с.1168G>T E390X	Anemia -diagnosed as DBA	Non	Decreased IgG level	Alive ,7y post haplo- HSCT

The patients in this series showed varying degree of BMF, transfusion dependency, dysplastic features and leukemia transformation. 3 developed hypoplastic MDS and one MDS/AML. Therefore, patients with MYSM1 mutation required careful follow up to detect any clonal evaluation. Our patients initially were transfusion dependent during infancy, then showed spontaneous improvement until age 9-10 years; they became transfusion depended, which may indicate clonal evolution as showed in our patients.

two of our cases missed diagnosed with DBA initially. MYSM1 may interact with the GATA1 pathway, essential for erythropoiesis.^9^ Patient with DBA often exhibit immunodeficiency, dysmorphic features, anemia, and thrombocytopenia.^16,17^ These findings suggest some DBA cases may be misdiagnosed, as hypoplastic anemia can occur in other rare diseases.

Five variants of MYSM1 have been reported in literatures figure (1) with 5 cases showed E390X and 2 cases R478X variant.^12^ MYSM1 mutations are associated with impaired DNA damage repair and chromatin remodeling, leading to hematopoietic failure.^5,6,18^ Activation of TP53 underlies hematopoietic manifestations of MYSM1 deficiency.^19^ The additional genetic aberrations identified in this series, such as TP53 mutation, monosomy, trisomy and CALR, underscore the heterogeneity of MYSM1-related disorders. These mutations may alter the clinical phenotype, impacting on disease severity and treatment outcome.

**Figure:**
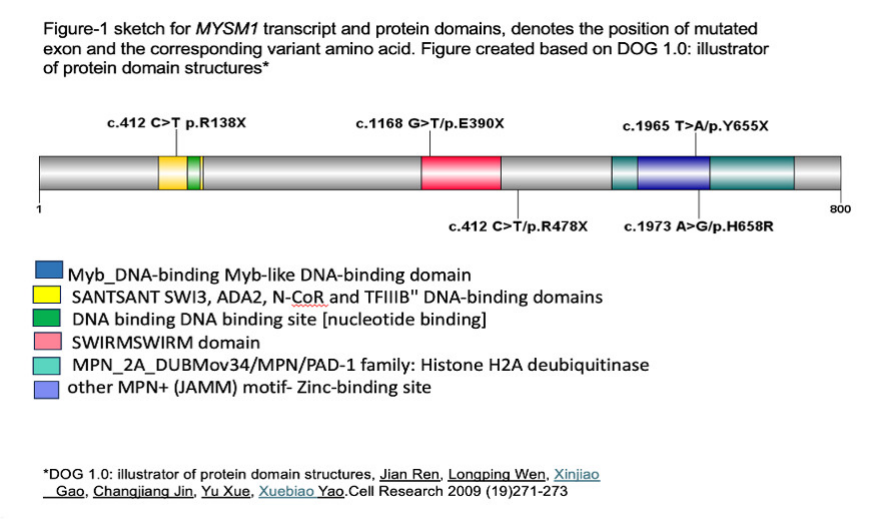


MYSM1 deficiency and IBMFS have in common the pathogenic mechanisms that involve instability of genome which leads to clonal hematopoiesis, MDS and AML. Chromatin remodeling abnormalities are believed to underlie the pathogenesis of MYSM1-related disorders while DNA repair defects are involved in the pathogenesis of some IBMFS. Both conditions cause chromosomal instability, cellular evolution and gene mutations including TP53, ASXL1 and RUNX1, although the frequency and the age of malignancy development may differ.^20,21^ In Fanconi anemia, AML is a known complication with a lifetime risk of 30%.^22,23^ About 40% of patients Dyskeratosis congenita developed malignancy in the middle age due to telomere dysfunction thus they are at high risk for MDS and AML.^23^ GATA2 defects lead to hematological malignancies in 70 % of patients by age 50 with a lifetime risk of AML and MDS of 32 % and 21 % respectively.^21^ For other conditions risk are varies ERCC6L2: The risk of AML/MDS is 10-30 % within 10 years^24,25^; SAMD9/SAMD9L: MDS is seen in 40-45 % of cases while AML is seen in 25-35 % of cases by 10 years of age.^26,27^ Shwachman-Diamond syndrome: The risk of AML is 20% by the age of 18 years; MECOM: The risk of myeloid malignancy is 5%; RUNX1: The lifetime risk of AML is 44%; CEBPA: It is seen that about 100% patients develop AML.^28–30^

progression to high-risk leukemia or MDS, or in situations of transfusion dependence highlighted the need for HSCT in IBMFS. Matched sibling donors (MSD) are preferred but unrelated donors or haploidentical donors and reduced intensity conditioning are now being used increasingly.^31^ Patients with MM with mutated TP53 who underwent hematopoietic stem cell transplantation had a poor outcome with a 3-year OS of 28%^25^

As of today, MM in patient with MYSM1 mutation has not been reported. Here we described four patients with MYSM1 mutation who developed MM for the first time. MYSM1 deficiency can also put patients at risk of other secondary cancers that are not related to the hematological system. literature shows that MYSM1 deficiency can lead to the susceptibility to solid tumors including skin cancer, gastrointestinal cancer, and genitourinary cancer due to the defective DNA repair and increased genomic instability^32–34^

Like IBMFS, MYSM1 individuals with mutations bear a high sensitivity to genotoxic stress due to their defective DNA damage response pathways. Hence, the conditioning regimens should be selected very carefully. The standard myeloablative conditioning leads to severe toxicity; therefore, reduced intensity conditioning (RIC) is used. RIC provides reduced genotoxic burden while providing adequate immunosuppression for grafting. As such, evidence from other conditions such as FA is in support of the use of RIC in reducing transplant related mortality and severe organ toxicity.^35^ RIC protocols which mainly involve the use of agents with low genotoxic potential such as fludarabine. But there is very little information regarding the clinical course of patients with MYSM1 deficiency. Such chemotherapies like busulfan or cyclophosphamide should be used with caution depending on the learnings from other bone marrow failure syndromes.

HSCT or the newly emerging gene therapy. HSCT is still the conventional treatment for MYSM1 related BMF and immunodeficiency; it holds the promise for lifesaving HSCT but with many challenges including graft versus host disease (GVHD) and complications from conditioning regimens. To the present moment, however, only a few patients have undergone transplantation.

Timing of HSCT has to be carefully balanced with extreme vulnerability for toxicities related to genetic defects and transplant-related complications. Reports identified 3 patients underwent MSD at the age of 23 Months,42 months and 6 years and were given fludarabine, treosulfan and alemtuzumab for the first two patients and Fludarabine based RIC for the last one.^8,36^ Two of our patients underwent haploidentical HSCT at 12 and 14 years of age and the follow up is 88 and 6 months respectively. All the patients engrafted successfully, one of which had chronic GVHD lung and eye. Thus, gene therapy has the potential to be an alternative remedy.

Data on gene therapy for IBMFS start to emerge, for DBA a RPS19 deficient CD34+ HSPC model was established using nanostraws in which EFS-RPS19 they used to restore erythroid differentiation and enhance erythropoiesis. For MYSM1 mutation the main idea is to correct the defect in MYSM1 in HSC ex vivo followed by autologous SCT. Hence, with the help of the current advancement in gene editing technologies like CRISPR-Cas9 it may be possible to make specific corrections with minimal chances of immune rejection and GVHD in the future.^37,38^ A patient with the homozygous c.1967A>G; p.His656Arg (H656R) MYSM1 variant showed spontaneous somatic genetic rescue which resulted in the hematopoietic improvement.^37^ This also supports the concept that in vivo gene therapy or the targeted correction in HSC for disorders that involve MYSM1 can be achieved, however this is still an emerging field. Managing the established success of HSCT with the advancement of gene therapies is still one of the major issues in the field.

### Conclusion

This case series has some important limitations, including a small number of patients and no long follow-up. However, it outlines the phenotypic and genetic complexity of MYSM1-associated BMFS and MDS. HSCT still represents the cornerstone of treatment with the possibility of achieving durable remission. Further studies are needed to delineate the complete spectrum of manifestations of MYSM1 mutation, best management options like gene therapy, and long-term outcomes. An integrated approach using molecular diagnosis may provide additional benefits for this rare genetic disorder.

### Authors’ Contribution

Conceptualization and writing original draft preparation: Alfadil Haroon Writing - review and editing: Syed Osman Ahmed, Chokri Ben Lamine, Mahmoud Aljurf, Hazzaa Alzahrani. Supervision: Mahmoud Aljurf, Hazzaa Alzahrani.

### Competing of Interest – COPE

Authors declare no conflict of interest.

### Ethical Conduct Approval – Helsinki – IACUC

Study has been approved by research ethical committee; data collected from bone marrow failure registry KFSH&RC-Riyadh RAC# 2021-084

### Informed Consent Statement

All authors and institution have confirmed this manuscript for publication.

### Data Availability Statement

All are available upon reasonable request from correspondence author.
